# Discrepancy between Cranial and DNA Data of Early Americans: Implications for American Peopling

**DOI:** 10.1371/journal.pone.0005746

**Published:** 2009-05-29

**Authors:** S. Ivan Perez, Valeria Bernal, Paula N. Gonzalez, Marina Sardi, Gustavo G. Politis

**Affiliations:** 1 CONICET, División Antropología, Facultad de Ciencias Naturales y Museo, Universidad Nacional de La Plata, La Plata, Buenos Aires, Argentina; 2 CONICET, División Arqueología, Facultad de Ciencias Naturales y Museo, Universidad Nacional de La Plata, La Plata, Buenos Aires, Argentina; University of Utah, United States of America

## Abstract

Currently, one of the major debates about the American peopling focuses on the number of populations that originated the biological diversity found in the continent during the Holocene. The studies of craniometric variation in American human remains dating from that period have shown morphological differences between the earliest settlers of the continent and some of the later Amerindian populations. This led some investigators to suggest that these groups—known as Paleomericans and Amerindians respectively—may have arisen from two biologically different populations. On the other hand, most DNA studies performed over extant and ancient populations suggest a single migration of a population from Northeast Asia. Comparing craniometric and mtDNA data of diachronic samples from East Central Argentina dated from 8,000 to 400 years BP, we show here that even when the oldest individuals display traits attributable to Paleoamerican crania, they present the same mtDNA haplogroups as later populations with Amerindian morphology. A possible explanation for these results could be that the craniofacial differentiation was a local phenomenon resulting from random (i.e. genetic drift) and non-random factors (e.g. selection and plasticity). Local processes of morphological differentiation in America are a probable scenario if we take into consideration the rapid peopling and the great ecological diversity of this continent; nevertheless we will discuss alternative explanations as well.

## Introduction

The biological diversity of South American human populations has been the focus of extensive research for more than a hundred years (see review in [1]). These investigations have been associated with intense interdisciplinary studies regarding the peopling of the Americas. The great interest in this subject is partially due to the fact that America was the latest continent colonized by modern humans (ca. 11,000–13,000 years B.P.; [2]) and also due to the high levels of morphological variation found in Native American populations. In this context, two main hypotheses have been proposed to account for this biological variation: a) the migratory hypothesis, which suggests that the biological variation among South American groups was the result of a variable number of migratory waves [3], [4]; and b) the local diversification hypothesis, i.e. that all South American groups descend from the same ancestral population or from populations related to each other, with local random (i.e. genetic drift) and non-random factors (i.e. selection and phenotypic plasticity) as the main causes of the diversification [5]–[7].

In recent years, the migratory hypothesis that postulates different biological origins for South American populations has received increased attention by researchers working with craniometric evidence [8]–[10]. This hypothesis, known as *two main biological components*, asserts that the morphological diversity of American human populations results from two successive migratory events. The first component, named Palaeoamericans, derived from Pleistocene Southeast Asian populations which expanded into America around 14,000 years BP. Morphologically they were characterized by long and narrow cranial vault (i.e. dolichocephalic morphology) and a narrow face. The second component, named Amerindians, from which most of modern American groups derive, corresponds to a migration of populations from Northeast Asia which occurred during the Early Holocene (ca. 8,000 years BP; [8]–[11]). These populations exhibited short and wide cranial vault, along with wide faces (i.e. brachycephalic morphology). In addition, it was pointed out that this Amerindian morphology corresponds with a mongoloid pattern of craniofacial shape. The presence of this cranial shape in America has been explained as the result of a “fixation” of the mongoloid morphology in North Asia, previous to the Amerindian migration.

In contrast, the molecular evidence available to date (i.e. mtDNA and nuclear DNA information) supports a single origin in Northeast Asia ca. 15,000 years BP for almost all American populations, followed by local diversification—probably with the exception of the Esquimo and Na-Dene groups [12]–[14]. Particularly, mtDNA studies have detected four major pan-American founding haplotypes (A2, B2, C1, D1), which are also frequent in Asia. In addition, other founding mtDNA haplotypes occur in the Americas, such as X2a, D2, and D3, which are found nearly exclusively in North America [12], [13]. The haplogroup distribution, together with the similar coalescence time for these haplotypes, has been used to support a single origin for extant American populations, as well as a swift pioneering process of the initial north to south migration [12]. In addition, coalescent analyses suggest an initial differentiation of the Northeast Asia populations, a bottleneck in Beringia ca. 20,000 years BP, ended with a population expansion in America ca. 15,000 years BP [12], [13].

The discrepancies between craniometric and molecular data, as well as the hypotheses supported by each kind of evidence, could be related either to the properties of both types of data, which provide different types of genealogical information, or to differences between the samples studied in each case. Particularly, quantitative traits and mtDNA differ in their respective mechanisms of inheritance (uniparental in mtDNA and biparental in quantitative traits), rate of change and degree of environmental influence [15]–[17]. On the other hand, the molecular data have been mainly obtained from extant or recent populations, whereas craniofacial variation has been assessed using skeletal samples from Early and Late Holocene populations. Hence, researchers who proposed the hypothesis of two main biological components assert that if the Paleoamericans did not survive or if their contribution to the biological variation of modern American populations was very small [7]–[9], the variation found among Later Late Holocene groups would not be relevant to discuss the early peopling

One way to approach this problem is by analyzing the cranial morphology of diachronic samples, ranging from Early to Late Holocene, for which ancient mtDNA data are also available. The few areas able to provide human remains dated as Early Holocene on the basis of ^14^C dates of human bones are [18]: East Central Brazil (Lagoa Santa, ca. 9,000–5,000 yr ^14^C BP; [10], [19]), the Bogotá savannah, Colombia (Tequendama, ca. 7,300–5,800 yr ^14^C BP; [20]) and the East Central Argentina (Arroyo Seco 2, ca. 7,800–6,300 yr ^14^C BP; [21]). However, East Central Argentina is the only region with a diachronic sequence ranging from 8,000 to 200 years BP [21], [22] for which both mtDNA and craniometric data are available. Even though this region holds important evidences, it has not yet been included in the discussion about the biological diversity of South American populations from a diachronic perspective. In this study we present the first analysis of a skeletal sample from East Central Argentina including both craniometric and molecular data. The goal of this work is to compare the pattern of temporal and spatial variation in both types of data and to discuss them in light of the current hypotheses about the peopling of America. The analysis of these data allows for a renewed approach to the problem of the biological diversity and peopling of this continent.

## Materials and Methods

### Samples

**Figure 1:**
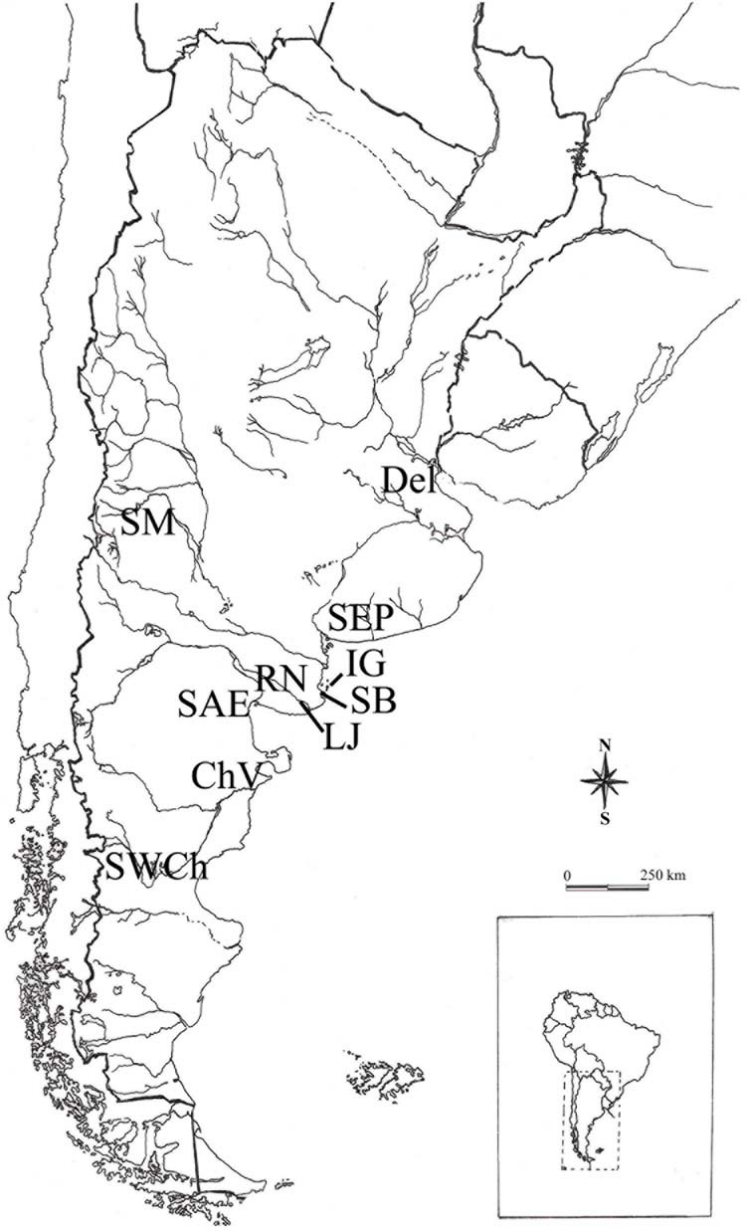
Map showing geographic location of the crania samples analyzed.

We studied the early site from East Central Argentina (i.e. Southeast of Pampa and Northeast of Patagonia, Fig. 1, Table 1) known as Arroyo Seco 2, dated between Late Pleistocene and Early/Middle Holocene—the human remains that were used here are dated on ca. 7,800–6,300 yr 14C BP [21], [23]—plus four samples of human remains corresponding to Middle and Earlier Late Holocene and four samples corresponding to Later Late Holocene from the same region. In addition, seven Late Holocene samples from neighbour regions were also analyzed (Table 1). All these samples include adult individuals of both sexes from hunter-gatherer groups, with presence of pottery in the Later Late Holocene.

**Table 1:** Sample composition, abbreviations, age, gender distribution and sample sizes.

Samples	Abbrev.	Region	Age*	F	M	Total
Southeast Pampa	SEP-emH#	Southeast Pampa	Early/Middle Holocene (ca. 7,800–6,300 years BP)	3	3	6
	SEP-elH	Southeast Pampa	Earlier Late Holocene (ca. 2,500–1,500 years BP)	2	7	9
	SEP-llH	Southeast Pampa	Later Late Holocene (ca. 1,500–200 years BP)	4	7	11
Isla Gama	IG-llH	Northeast Patagonia	Later Late Holocene (ca. 1,500–200 years BP)	7	5	12
San Blas	SB-llH	Northeast Patagonia	Later Late Holocene (ca. 1,500–200 years BP)	15	18	33
Laguna del Juncal	LJ-elH#	Northeast Patagonia	Earlier Late Holocene (ca. 3,500–2,500 years BP)	12	19	31
Negro River Valley	RN-elH1	Northeast Patagonia	Earlier Late Holocene (ca. 3,500–2,500 years BP)	13	10	23
	RN-elH2	Northeast Patagonia	Earlier Late Holocene (ca. 2,500–1,500 years BP)	2	8	10
	RN-llH	Northeast Patagonia	Later Late Holocene (ca. 1,500–200 years BP)	9	12	21
San Antonio Este	SAE-llH	Centre Patagonia	Later Late Holocene (ca. 1,500–200 years BP)	3	5	8
Chubut River Valley	ChV-elH	Centre Patagonia	Earlier Late Holocene (ca. 2,500–1,500 years BP)	6	10	16
	ChV-llH	Centre Patagonia	Later Late Holocene (ca. 1,500–200 years BP)	18	20	38
Southwest Chubut	SWCh-llH	Centre Patagonia	Later Late Holocene (ca. 1,500–200 years BP)	7	7	14
South Mendoza	SM-elH	Northwest Patagonia	Earlier Late Holocene (ca. 2,500–1,500 years BP)	8	15	23
	SM-llH	Northwest Patagonia	Later Late Holocene (ca. 1,500–200 years BP)	6	9	15
Delta of Parana	Del-llH	Northeast Pampa	Later Late Holocene (ca. 1,500–200 years BP)	5	8	13
Total						283

* Approximate sample ages according to radiocarbon dating obtained from human bones and contextual information.

# Samples characterized molecularly.

The Arroyo Seco 2 archaeological site presents exceptional evidence to study the early peopling of America [23]. This multi-component open-air site is dated from 12,500 ^14^C yr BP to the XIX Century [24] and nowadays is located at about 50 km north from the Atlantic Coast in the Buenos Aires Province of Argentina (38°21′ lat S. and 60°14′ lon W). Arroyo Seco 2 has an early component containing a lithic assemblage of unifacial, marginally retouched tools associated with bone remains of guanaco (camelid), Pampean deer, and nine extinct megafauna: *Paleolama*, *Equus*, *Hippidion*, *Toxodon*, *Megatherium*, *Eutatus*, *Glossotherium*, *Macrauchenia*, and *Glyptodon* [23]. Apart from this early component, the site contains one of the best records of human remains for the Early/Middle Holocene transition in South America. To date, 45 human skeletons have been uncovered and there are 21 dates from ca. 7,800 to 4,500 ^14^C yr BP related to them [23]. The span of dates from the primary and secondary burials of Arroyo Seco 2, suggests the use of the site—not continuously but redundantly—for inhumations purposes, for more than 3,000 years during the Early and Middle Holocene.

Middle and Earlier Late Holocene samples from East Central Argentina contain individuals of different sites from Laguna del Juncal archaeological locality (Laguna del Juncal, Río Negro Valley 1 and 2; see Table 1), placed south from Viedma city in the Río Negro Province of Argentina (40°48′ lat S. and 62°58′ lon W), and one sample from Southeast Pampa (Table 1). The samples from East Central Argentina dated on Later Late Holocene come from various archaeological sites from Río Negro, placed near Laguna del Juncal and Peninsula San Blas (40°33′ lat S. and 62°13′ lon W), and the Buenos Aires Province (Table 1).

Specimens are housed at División Antropología of the Museo de La Plata, Museo Etnográfico ‘J. B. Ambrosetti’ in Buenos Aires and INCUAPA in Olavarria, Argentina.

### Preliminary analyses

Because most samples are sex balanced, males and females were pooled in the analyses to obtain a greater sample size. In order to control some sources of variation related to sex, we analyzed size standardized adult individuals of both sexes. The observational error was controlled using the experimental design introduced by Perez [1]. The results showed that photographing and digitalization of landmarks and semilandmarks procedures did not generate significant observational error [1].

### Morphometric analyses

**Figure 2:**
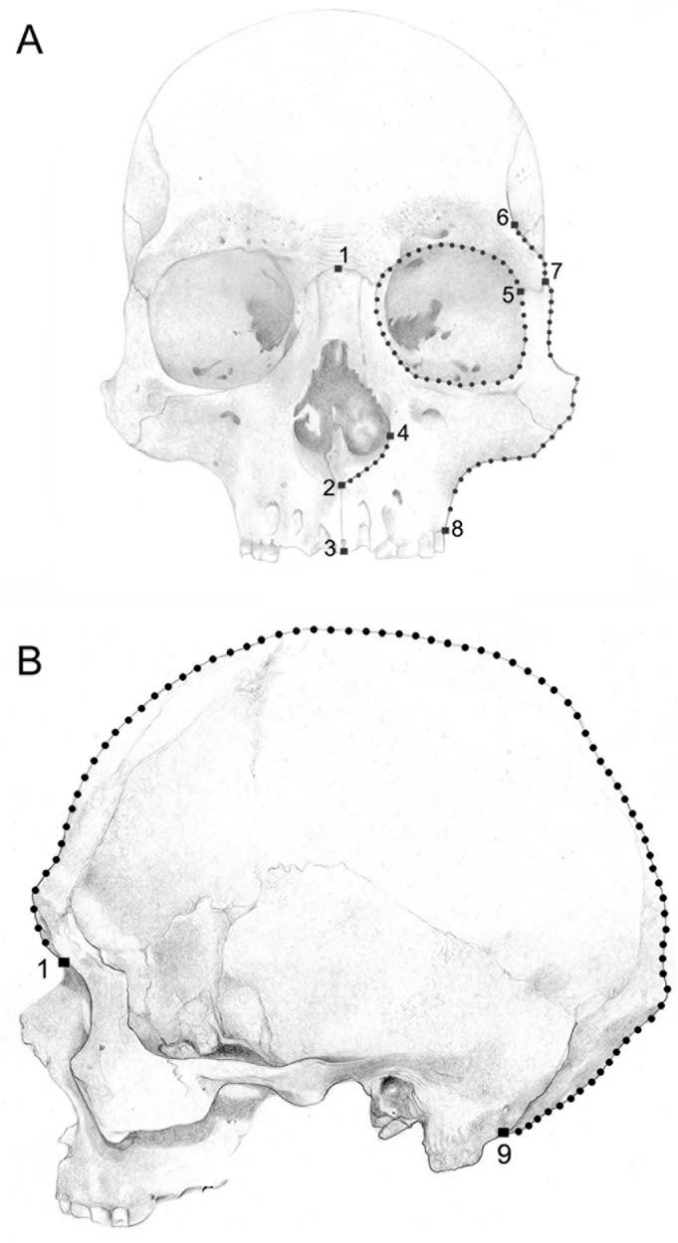
Allocated geometric coordinates are displayed with different symbols. Landmarks are represented as squares (▪), whereas semilandmarks are represented as circles (•) on face (A) and vault (B) views. The numbers correspond to the following landmarks: nasion (1); nasospinale (2); prosthion (3); alare (4); ectoconchion (5); frontotemporale (6); frontomalare temporale (7); ectomolare (8); post-mastoid (9).

The craniofacial variation was analyzed with geometric morphometrics techniques [25], [26] employing an arrangement of two-dimensional coordinates of biologically definable landmarks and semilandmarks (Fig. 2). Most comparisons were done on the facial skeleton, which is not affected by the cranial deformation present in these samples. We also performed an analysis of vault morphology in non-deformed skulls. Specimens were photographed with an Olympus SP 350 digital camera with the skull positioned according to the Frankfurt plane. For facial images, the camera lens was located in the coronal plane [27] and digital images were obtained from the crania in frontal view. Facial images were taken at 250 mm from the prosthion point. Eight landmarks and seventy-four semilandmarks (Fig. 2A) were obtained from the facial skeleton. For vault skeleton, digital images were obtained from the crania in lateral (left side) view. Lateral view images were taken at 300 mm from the Euryon. Coordinates for two landmarks and seventy-eight semilandmarks were recorded on the lateral view of the crania (Fig. 2B). The landmarks were located following the definitions of Buikstra and Ubelaker [27]. The application MakeFan6 [28], which places alignment ‘fans’ at equal angular displacements along a curve, was used to ensure consistent placement of the craniofacial semilandmark coordinates. Both landmarks and semilandmarks were afterwards digitized by one of us (SIP) using tpsDIG 1.40 software [29].

In geometric morphometrics, shape variation can be defined as the information that remains in the coordinates of landmarks and semilandmarks after the differences due to location, scale and orientation (i.e. non-shape differences) have been removed [25]. To eliminate non-shape variation in such coordinates—by overlaying them according to a least-square optimization criterion—the superimposition method known as Generalized Procrustes Analysis was used [25], [26]. At the start the coordinates of any single individual are centered at the origin (0,0) by substracting the centroid or mean location of all landmarks and semilandmarks. After that, the centroid size of the configuration (the square root of the summed square distance of all landmarks from the centroid) is set to 1 dividing the coordinates by the initial centroid size of the individual. An iterative procedure is used to determine the mean form onto which all individuals are aligned. To do so, all individuals are first aligned as a single individual, and their mean shape is calculated. All individuals are then rotated to minimize the added squared differences of point coordinates between each one of them and the estimated mean shape or reference form. This procedure is repeated until the mean shape does not change substantially after iteration of the orientation procedure. At this point, the individuals are in partial Procrustes superimposition onto the reference form [25], [26]. When outlines are digitized as discrete points (i.e. semilandmarks), a step is added to the Generalized Procrustes Analysis to minimize the variation tangential to the curve, since individual curve points are not claimed to be homologous in all subjects. Consequently, the variation along tangent directions is not informative, and only the coordinate normal to the outline bears information about differences between individuals or groups. We aligned semilandmarks by means of perpendicular projection or minimum Procrustes distance criteria [26], [30]. In addition to optimally translating, scaling, and rotating landmarks, the semilandmarks are slid along the outline curve until they match as much as possible the positions of corresponding points along the outline of the reference individual [25], [30], minimizing the Procrustes distance between the subject and the reference individual.

Shape differences among samples and individuals were studied using the aligned coordinates. These coordinates were used to perform a Principal Component or Relative Warp Analysis (RW) to describe major trends in shape among samples [25], [26]. An important aspect of this analysis is that variation along the relative warp axes can be expressed as intuitive deformation grid diagrams showing the difference from the mean form or reference. tpsRelw 1.44 [29] was used to perform the geometric morphometric analyses.

The pattern of ordination produced by the first two relative warps of the facial data was compared with temporal and geographical differences among the samples using the PROTEST analysis [31]. To describe such differences we created an ordination matrix with radiocarbon dating and geographic coordinates (i.e. latitude and longitude) for each sample. PROTEST analysis compare this ordination by using the sum of the squared residuals between ordinations in their optimal superimposition such as a measurement of association (*m_12_*; [31]). There are several strategies for superimposition, but the Generalized Procrustes Analysis used in geometric morphometrics is the simplest approach (see above; [31]). A permutation procedure (10,000 permutations) was used afterwards to assess the statistical significance of the Procrustean fit. PROTEST analysis was performed using vegan 1.8–8 package for R 2.6.1 [32].

### Molecular data

mtDNA haplogroups for some of the East Central Argentina samples have previously been obtained in different works [33]–[36]. aDNA analytical methods used by Lalueza et al. [33] and Figueiro and Sans [36; Figueiro personal communication] are similar. Teeth and well-preserved bone pieces were handled under stringent precautionary measures to prevent extraneous contaminations. Teeth were sequentially soaked in 15% or 20% HCl for 10 min to remove dirt and carbonate deposits (in addition, Lalueza et al. [33] employed 70% ethanol for 10 min and rinsed in sterile double-distilled water for 30 min). Subsequently, teeth and bones were irradiated with UV lamp for 15 min. Next, the external surface of the samples was removed using a sand-blaster to eliminate both soil and exogenous DNA contaminants. Samples were powdered under liquid nitrogen in a Spex freezer mill fitted with UV-sterilized tubes and impactors. The obtained powder was used to DNA extraction, employing a standard, high-volume phenol/chloroform protocol [33]. Several strategies were strictly followed with the object to demonstrate authenticity of the obtained data. All analyses were performed in laboratories exclusively dedicated to ancient DNA manipulation. To trace possible contamination, mtDNA sequences from the authors and other laboratory members who had manipulated the bones were obtained. To characterize the mtDNA lineages, DNA purified from bone and teeth was amplified by PCR using specific primers [33], [35], [36]. After amplification, the mtDNA products were classified with the specific endonucleases defining each Amerindian haplogroup and then electrophoresed on agarose gels. In addition, several samples that yielded significant PCR amplification products for the HVRI mtDNA region were used for further mtDNA sequencing characterization [33], [35].

## Results

**Figure 3:**
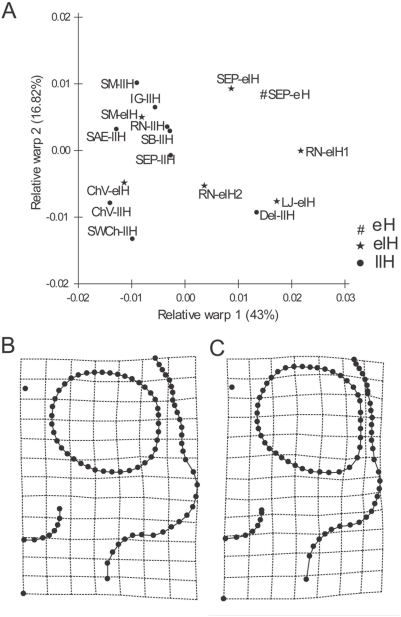
Relative Warp analysis of the face. A) Ordination of the 16 East Central samples in the space of the first two relative warps, based on the partial warp and uniform component variables calculated for the face. The circles (•) represent the consensus individual or mean shape for each Later Late Holocene sample (llH), stars (★) represent the consensus individual for each Earlier Late Holocene sample (elH), and number sign (#) represents the consensus individual for the Early Holocene sample from Arroyo Seco 2 (eH). B and C) Facial shape changes implied by variation along the first relative warp axis is shown as deformation grids. Grids show shape changes for negative (B) and positive (C) deviations from the mean for RW1.

**Figure 4:**
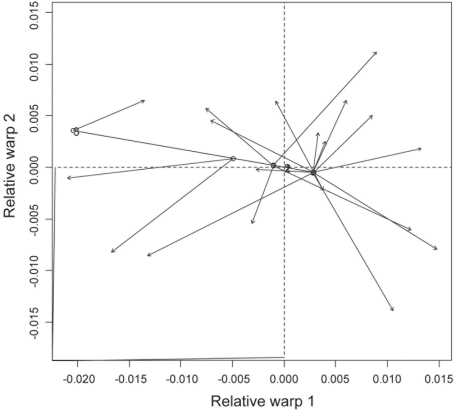
Procrustes fit of geographic coordinates and temporal data (circle) onto the position of mean facial shape for each East Central Argentina sample on relative warps 1 and 2.

**Figure 5:**
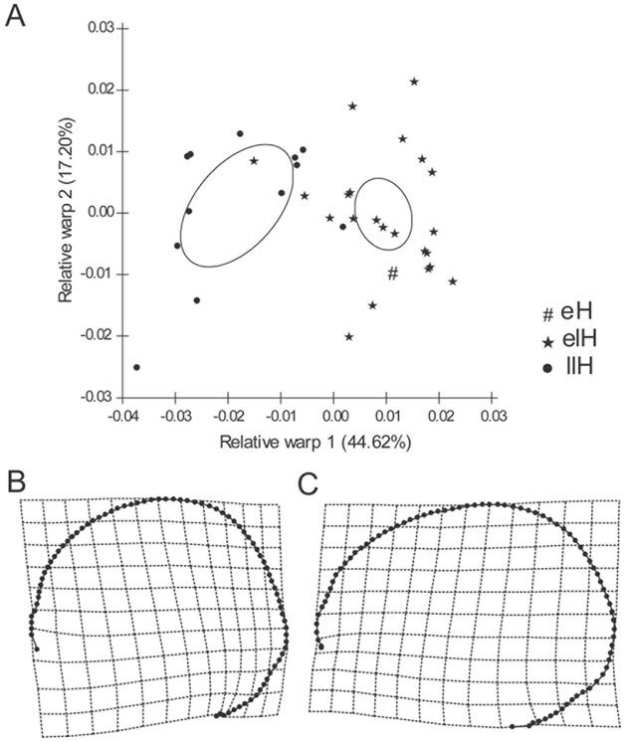
Relative Warp analysis of the cranial vault. A) Ordination of the individuals from East Central samples in the space of the first two relative warps, based on the partial warp and uniform component variables calculated for the vault. The circles (•) represent the individuals for Later Late Holocene sample (llH), stars (★) represent the individuals for each Earlier Late Holocene sample (elH), and number sign (#) represents the individual for the Early Holocene sample from Arroyo Seco 2 (eH). The ellipses are the 95% confidence intervals of Earlier and Later Late Holocene mean samples. B and C) Vault shape changes implied by variation along the first relative warp axis is shown as deformation grids. Grids show shape changes for negative (B) and positive (C) deviations from the mean for RW1.

For the facial skeleton, the relative warp 1 shows that the samples from Arroyo Seco 2 and the four samples corresponding to the Middle and Earlier Late Holocene from East Central Argentina separate themselves from the four Later Late Holocene samples of the same region (Fig. 3A). Almost all samples of the neighbour regions—with exception of the Delta of Parana sample—have a similar shape to the Later Late Holocene samples from East Central Argentina (Fig. 3A). Figures 3B and 3C display the deformation grids for these data, showing that the main differences along the first axis are located in the orbital and zygomatic shape, as well as in the relative size of the orbit. Particularly, the Later Late Holocene samples show the widest facial skeleton, with wider malar bones, and relatively smaller orbits. The Procrustes analysis confirm this diachronic pattern of differences (Fig. 4), showing a significant association between facial shape and temporal plus geographic dimension (*m_12_* = 0.447, *P* = 0.016), being both, temporal and geographic variation, important to explain facial shape differences (temporal variation *m_12_* = 0.446, *P* = 0.016; geographic variation *m_12_* = 0.489, *P* = 0.012). The relative warp 1 of vault variation indicates that the individuals from Arroyo Seco 2 and those of the Middle and Earlier Late Holocene from East Central Argentina (mainly the sample from Laguna del Juncal) are different from the Later Late Holocene individuals of the same region (Fig. 5A). Figures 5B and 5C display the deformation grids for these data, showing that the earlier samples have longer, or dolicocephalic, cranial vault. This craniometric variation, particularly in the relative cranial length and facial width, seems to be consistent with the pattern of morphological variation interpreted as differences between Paleoamerican and Amerindian groups [9].

The ancient mtDNA analyses of individuals from Arroyo Seco 2 and Laguna del Juncal samples, however, show the presence of native American haplogroups B, C and D [33], [35], [36]. This agrees with the main incidence of these three haplogroups in mtDNA sequences of recent populations from the same region [33], [35], [36], [37], related to our Later Late Holocene samples. Figueiro and Sans [36] successfully recover DNA from 8 individuals from a total of 23 individuals studied in Arroyo Seco 2 site. The haplogroups B (n = 3; 37.5%), C (n = 4; 50%) and D (n = 1; 12.5%) were found [36]. In Laguna del Juncal haplogroups C (n = 4; 26.7%) and D (n = 11; 73.3%) are present [33; but note that this sample sizes represent the haplogroups from Aonikenk plus Laguna del Juncal site]. In addition, several samples of haplogroups C and D were successfully cloned and sequenced by Lalueza et al. [33] and Garcia-Bour et al. [35], verifying the results provided by the analysis of restriction site polymorphisms. The haplogroup frequencies from Arroyo Seco 2 and Laguna del Juncal are very similar to the haplogroup frequencies from recent groups of Central Argentina. Particularly, a recent population from Pampa, the Mapuche, has values of A = 6.14%, B = 35.96%, C = 23.9% and D = 34% [38] and a recent population from Northwest Patagonia, the Pewenche, has values of A = 2%, B = 9%, C = 37% and D = 52% [39]. Such values match with the expected clinal change of haplogroups frequencies from north to south observed in South America [34], [37], [39].

## Discussion

The results obtained show that morphological variation in East Central Argentina does not correlate with mtDNA differences. The oldest samples from the region under study, dated on ca. 8,000–2,000 years BP, present more elongated crania than the Later Late Holocene samples, but both groups have the same mtDNA haplogroups (and even haplotypes). It was pointed out that mtDNA variation in modern Native Americans support a single expansion into America of groups from Northeast or Central Asia [12]–[14], [37]. Conversely, the same morphological differences were also observed in other regions of South America and have been used as evidence of different migratory waves, according with the two main biological components hypothesis, with a major population replacement taking place around 8,000–3,000 years BP [8]–[10].

This hypothesis asserts, in particular, that Amerindians have a mongoloid craniofacial shape, while Paleoamericans have a generalized morphology. Although mongoloid phenotypic pattern encompasses highly variable groups, most populations of North East Asia share two phenotypic traits: facial flatness [40] and the synodont dental pattern [41], [42]. However, despite the two main biological components affirmation, facial flatness is absent among Late Holocene South American groups [40], [43]. In addition, several studies that analyze craniofacial similarities between American groups and other worldwide populations demonstrate that modern aborigines do not present the typical morphology of North East Asia [44], [45]. Specifically, “the American groups are more apt to join Europeans than Asiatics” [44], suggesting that the Late Holocene South American groups have not specific mongoloid craniofacial traits.

The lack of concordance between molecular data and craniofacial morphology has also been observed when studying the groups who inhabited the southernmost part of America during historic times [1]. The Fueguian groups have been classified as Paleoamericans by cranial shape (i.e. high levels of dolicocephaly and robusticity), and differ morphologically from other South American groups with brachycephalic morphology (i.e. Amerindian morphology sensu Neves and co-workers) [1]. However, the molecular studies show that they carried Native American mtDNA haplotypes C and D [34]–[35]. In addition, the study of Y-STRs sequences shows similar results [35], suggesting that the Fueguians are close to Amerindian populations from South Central Chile and Argentina.

Different hypotheses can be suggested to explain the discrepancy between mtDNA and craniometrical variation in South America. Because mtDNA is essentially a single locus, it could have been subject to considerable genetic drift, even more than morphological traits [7], during the Pleistocene-Early Holocene. Particularly, some North American aDNA studies suggest that the founding migrants exhibited greater molecular diversity than what has been previously recognized, showing that during the Early-Middle Holocene there were more than five founding mtDNA lineages [46], [47]. If the hypothetical Paleoamerican component had a particular mtDNA variation, it could have been modified during the initial South American peopling by founder effect. As Paleoamericans moved south, new territories were colonized by small groups carrying a subsample of haplotypes from the ancestral populations. Therefore, some Paleoamerican specific haplotypes could have gotten lost in Southern South America as the consequence of this process [34], [37], [39].

An alternative hypothesis is the existence of a selective sweep in mtDNA variation. Some investigators have pointed out that mtDNA variation has been influenced by climatic selection related to heat generation [48]. According to these authors some haplogroups, that cause lowered coupling efficiency generating less ATP and more heat, were positively selected during the radiation of modern humans into colder climates. Hence, natural selection might have favored certain mtDNA haplogroups when the Asian groups migrated into colder climates in Northeast Asia and peopled America through Beringia. Assuming that the haplogroups found in America were positively selected, their presence in Paleoamericans and Amerindians could be explained by convergent evolution. However, several recent papers found results contrary to what this hypothesis predicted, and support random genetic drift as the main factor in shaping mtDNA variation [49], [50].

Alternatively, if we consider the hypothesis of two main biological components, and a major replacement of Paleoamericans by Amerindians during the Middle or Earlier Late Holocene, the “invading” population (i.e. Amerindians) could have had a genetic exchange with the local population (i.e. Paleoamericans). Currat and co-workers [51]–[53] showed that even the presence of low values of genetic exchange between local and “invading” populations can result in a major contribution of local neutral genes into the invader gene pool, and almost exclusively in this direction. This is important here because even if the Paleoamericans were replaced by a population of Amerindian morphology, mtDNA variation found in extant populations (of Amerindian morphology) could have been originated in Paleoamerican populations. These arguments contrast with the common suggestion that if a major replacement of Paleoamericans by Amerindians occurred during the Middle or Earlier Late Holocene [7]–[9], the variation among Later Late Holocene groups would not be relevant to discuss the early peopling.

Finally, we suggest that the lack of concordance between molecular evidence and morphological data could be explained if we take into consideration that craniofacial variation among human populations could mainly result from the action of non-random factors such as directional selection and phenotypic plasticity. These processes are suggested by the correspondence between craniofacial morphology and ecological variables (i.e. diet and climate) in South American samples. Specifically, Perez and Monterio [54] found a strong correspondence between brachycephalic crania and farmer groups, while the dolicocephalic ones associated with hunter-gatherers. If we take into account that all samples assigned as Paleoamericans belong to hunter-gatherers, and that changes in diet (i.e. production of domesticated resources) and food preparation technology (i.e. pottery and grinding use) took place between 8,000 and 2,000 years BP [55], [56], the influence of ecological variables could be important to explain the morphological differences between Paleoamericans and Amerindians. A similar trend of change in craniofacial shape—mainly in the facial shape, but also in the cranial vault—has been associated with ecological factors in other world regions. Such factors include differences in the diet or masticatory activities related to the economies of hunter-gatherers vs. farmers [57]–[61]. The importance of environment-dependent phenotype expression during the ontogeny has been suggested to explain this morphological variation. Directional selection and/or phenotypic plasticity can generate fast morphological changes and account for the craniofacial variation found among American populations [7], [54]. All this stresses the importance of elucidating the probable sources of variation of craniofacial morphology, including random and non-random factors, before being able to affirm that different traits reflect different ancestry.

Although we need more studies to discuss the alternative hypotheses about the discrepancy between mtDNA and craniometrical variation in South America, other molecular analyses suggest that such discrepancies could be mainly the result of non-random factors acting over the morphological divergence. Specifically, the Native American Y chromosome haplogroups are also originated in Central Asia and share similar coalescent dates, indicating that they have a single ancestral gene pool [12]–[14], [37]. The American groups also share alleles at specific microsatellite loci that are not found in any Old World populations [14]. Therefore, the chromosomical and microsatellite loci molecular studies, along with the evidence that the Early Holocene samples with Paleoamerican cranial morphology carried the same mtDNA haplogroups as modern Amerindians, suggest that the Holocene East Central Argentina human populations did not have two different extra-American origins but a single one in Central or Northeast Asia.

In light of the results that were discussed here, the craniometric variation found in samples from other South American regions also dated ca. 8,000 to 5,000 years BP [18], such as the Bogotá savannah in Colombia [9] and Lagoa Santa in Brazil [8], should be carefully interpreted. This is particularly true for the samples from the Bogotá savannah, with radiocarbon dates performed on human bones similar to Arroyo Seco 2 [18], and displaying morphologies attributable to Paleoamericans. The craniofacial comparison of these samples with samples displaying Amerindian like morphologies—as those assigned to Later Late Holocene from Perú—has been used up till now as evidence supporting the existence of two main biological components in the peopling of America [9]. However, craniofacial morphology in these regions could be also related to non-random factors, since the diachronic samples compared (e.g. Tequendama or Lagoa Santa vs. Peru sample; [8], [9]) do not belong to the same area but come from regions not only geographically distant but also ecologically different (i.e. hunter-gatherers vs. farmer groups respectively).

The analysis of processes and events of population diversification involved in the American peopling has proven to be very difficult but could be addressed through the study of regional population histories, integrating diachronic skeletal samples with chronological control, mtDNA data of ancient and extant populations, archaeological records and ecological information, as well as quantitative descriptions of morphological variation. Following this approach we show here that even when the oldest samples display traits attributable to Paleoamerican crania, they present the same mtDNA haplogroups as later populations with Amerindian morphology. A possible explanation for these results could be that the craniofacial differentiation was a local phenomenon resulting from random (i.e. genetic drift) and non-random factors (e.g. selection and plasticity). Local processes of morphological differentiation in America are a probable scenario if we take into consideration the rapid peopling and the great ecological diversity of this continent.

## References

[1] Perez SI, Bernal V, Gonzalez PN (2007). Morphological differentiation of aboriginal human populations from Tierra del Fuego (Patagonia): implications for South American peopling.. Am J Phys Anthropol.

[2] Borrero LA (1999). The prehistoric exploration and colonization of Fuego-Patagonia.. J World Prehistory.

[3] Imbelloni J (1938). Tabla clasificatoria de los indios. Regiones biológicas y grupos raciales humanos de América.. Physis.

[4] Neves WA, Pucciarelli HM (1989). Extra-continental biological relationships of early South American human remains: a multivariate analysis.. Cien Cult.

[5] Hrdlicka A (1912). Early Man in South America..

[6] Greenberg J, Turner CG, Zegura SL (1986). The settlement of the Americas: a comparison of the linguistic, dental and genetic evidence.. Curr Anthrop.

[7] Powell JF, Neves WA (1999). Craniofacial morphology of the first Americans: pattern and process in the peopling of the New World.. Yearb Phys Anthropol.

[8] Neves WA, Prous A, González-José R, Kipnis R, Powell J (2003). Early Holocene human skeletal remains from Santana do Riacho, Brazil: implications for the settlement of the New World.. J Hum Evol.

[9] Neves WA, Hubbe M, Correal G (2007). Human skeletal remains from Sabana de Bogotá, Colombia: a case of Paleoamerican morphology late survival in South America?. Am J Phys Anthropol.

[10] Neves WA, Hubbe M (2005). Cranial morphology of early Americans from Lagoa Santa, Brazil: Implications for the settlement of the New World.. Proc Natl Acad Sci USA.

[11] González-José R, González-Martín A, Hernández M, Pucciarelli HM, Sardi M (2003). Craniometric evidence for Palaeoamerican survival in Baja California.. Nature.

[12] Tamm E, Kivisild T, Reidla M, Metspalu M, Smith DG (2007). Beringian standstill and spread of Native American founders.. PLoS ONE.

[13] Fagundes NJR, Kanitz R, Eckert R, Valls ACS, Bogo MR (2008). Mitochondrial population genomics supports a single pre-Clovis origin with a coastal route for the peopling of the Americas.. Am J Hum Genet.

[14] Goebel T, Waters MR, O'Rourke DH (2008). The Late Pleistocene dispersal of modern humans in the Americas.. Science.

[15] Brown WM, Prager EM, Wang A, Wilson AC (1979). Rapid evolution of animal mitochondrial DNA.. Proc Natl Acad Sci USA.

[16] Cavalli-Sforza LL, Menozzi P, Piazza A (1994). The history and geography of human genes.

[17] Jobling MA, Tyler-Smith C (2003). The human Y chromosome: an evolutionary marker comes of age.. Nat Rev Genet.

[18] Politis G, Prates L, Perez SI (2009). Los primeros Americanos. Una historia arqueológica y bioantropológica del poblamiento de América.

[19] Dillehay TD (2000). The Settlement of the Americas: A New Prehistory.

[20] Correal Urrego G, van der Hammen T (1977). Investigaciones arqueológicasen los abrigos rocosos del Tequendana.

[21] Scabuzzo C, Politis G (2007). Early-Holocene secundary burials in the Pampas of Argentina.. Curr Res Pleistocene.

[22] Bernal V, Gonzalez P, Perez SI, Pucciarelli HM (2008). Entierros humanos del Noreste de Patagonia: nuevos fechados radiocarbónicos.. Magallania.

[23] Politis G, Gutierrez MA, Scabuzzo C (2009). Estado actual de las investigaciones en el sitio 2 de Arroyo Seco (región pampeana, Argentina).

[24] Steele J, Politis G (2009). AMS 14C dating of early human occupation of southern South America.. J Arch Sc.

[25] Adams DC, Rohlf FJ, Slice DE (2004). Geometric morphometrics: ten years of progress following the ‘revolution’.. Ital J Zool.

[26] Zelditch ML, Swiderski DL, Sheets HD, Fink WL (2004). Geometric morphometric for Biologists: a primer.

[27] Buikstra J, Ubelaker D (1994). Standards for data collection from human skeletal remains.

[28] Sheets HD (2003). IMP-Integrated Morphometrics Package.

[29] Rohlf FJ (2008). Tps Serie Softwares.

[30] Perez SI, Bernal V, Gonzalez P (2006). Differences between sliding semilandmarks methods: implications for shape analyses of human populations.. J Anat.

[31] Peres-Neto PR, Jackson DA (2001). How well do multivariate data sets match? The advantages of a Procrustean superimposition approach over the Mantel test.. Oecologia.

[32] R Development Core Team (2008). R: a language and environment for statistical computing.

[33] Lalueza C, Perez-Perez A, Prats E, Cornudella L, Turbon D (1997). Lack of founding Amerindian mitochondrial DNA lineages in extinct aborigines from Tierra de Fuego-Patagonia.. Hum Mol Genet.

[34] Moraga LM, Rocco P, Miquel JF, Nervi F, Llop E (2000). Mitochondrial DNA polymorphisms in Chilean aboriginal populations: implications for the peopling of the southern cone of the continent.. Am J Phys Anthropol.

[35] Garcıa-Bour J, Perez-Perez A, Alvarez S, Fernandez A, Lopez-Parra AM (2004). Early population differentiation in extinct aborigines from Tierra del Fuego-Patagonia: ancient mtDNA sequences and Y-chromosome STR characterization.. Am J Phys Anthropol.

[36] Figueiro G, Sans M (2007). Primeros resultados del análisis de ADN mitocondrial del sitio Arroyo Seco 2, Provincia de Buenos Aires, Argentina.. Rev Arg Antrop Biol.

[37] Schurr TG (2004). The peopling of the new world: perspectives from molecular anthropology.. Annu Rev Anthropol.

[38] Bailliet G, Rothhammer F, Carnese FR, Bravi CM, Bianchi NO (1994). Founder mitochondrial haplotypes in Amerindian populations.. Am J Hum Genet.

[39] Merriwether DA, Rothhammer F, Ferrell RE (1995). Distribution of the four lineage haplotypes in Native Americans suggests a single wave of migration for the New World.. Am J Phys Anthropol.

[40] Hanihara T (2000). Frontal and facial flatness of mahor human populations.. Am J Phys Anthropol.

[41] Turner CG (1984). Advances in the dental search for native american origins.. Acta Anthropogenet.

[42] Turner CG (1987). Late Pleistocene and Holocene population history of East Asia based on dental variation.. Am J Phys Anthropol.

[43] Sardi ML, Pucciarelli HM, Dahinten SL (2001). Evaluación de la mongolización en amerindios.. Rev Arg Antrop Biol.

[44] Howells WW (1989). Skull shape and the map. Papers of the Peabody Museum of Archaeology and Ethnology.

[45] Brace CL, Nelson AR, Seguchi N, Oe H, Sering L (2001). Old World sources of the first New World human inhabitants: A comparative craniofacial view.. Proc Natl Acad Sci USA.

[46] Kemp BM, Malhi RS, McDonough J, Bolnick DA, Eshleman JA (2007). Genetic analysis of Early Holocene skeletal remains from Alaska and its implications for the settlement of the Americas.. Am J Phys Anthropol.

[47] Malhi RS, Kemp BM, Eshleman JA, Cybulski J, Smith DG (2007). Mitochondrial haplogroup M discovered in prehistoric North Americans.. J Archaeol Sci.

[48] Ruiz-Pesini E, Mishmar D, Brandon M, Procaccio V, Wallace DC (2004). Effects of purifying and adaptive selection on regional variation in human mtDNA.. Science.

[49] Sun C, Kong Q, Zhang Y (2006). The role of climate in human mitochondrial DNA evolution: A reappraisal.. Genomics.

[50] Amo T, Brand MD (2007). Were inefficient mitochondrial haplogroups selected during migrations of modern humans? A test using modular kinetic analysis of coupling in mitochondria from cybrid cell lines.. Biochem J.

[51] Currat M, Excoffier L (2004). Modern humans did not admix with Neanderthals during their range expansion into Europe.. PLoS Biol.

[52] Currat M, Excoffier L (2005). The effect of the Neolithic expansion on European molecular diversity.. Proc R Soc Lond B.

[53] Currat M, Ruedi M, Petit RJ, Excoffier L (2008). The hidden side of invasions: Massive introgression by local genes.. Evolution.

[54] Perez SI, Monteiro LR (2009). Non-random factors in modern human morphological diversification: a study of craniofacial variation in southern South American populations.. Evolution Int J Org Evolution.

[55] Price TD (2009). Ancient farming in eastern North America.. Proc Natl Acad Sci USA.

[56] Berberián EE, Nielsen AE (2001). Historia Argentina prehispánica.

[57] Larsen CS (2006). The agricultural revolution as environmental catastrophe: Implications for health and lifestyle in the Holocene.. Quat Internat.

[58] van Vark GN, Kuizenga D, L'Engle Williams F (2003). Kennewick and Luzia: lessons from the European Upper Paleolithic.. Am J Phys Anthropol.

[59] Sardi ML, Novellino PS, Pucciarelli HM (2006). Craniofacial morphology in the Argentine Center-West: Consequences of the transition to food production.. Am J Phys Anthropol.

[60] Stynder DD, Ackermann RR, Sealy JC (2007). Craniofacial variation and population continuity during the South African Holocene.. Am J Phys Anthropol.

[61] Sardi ML, Ramírez-Rozzi F, Pucciarelli HM (2004). The Neolithic transition in Europe and North Africa. The functional craneology contribution.. Anthropol Anz.

